# Patent Ductus Arteriosus in Preterm Infants: Do We Have the Right Answers?

**DOI:** 10.1155/2013/676192

**Published:** 2013-12-23

**Authors:** Hesham Abdel-Hady, Nehad Nasef, Abd Elazeez Shabaan, Islam Nour

**Affiliations:** Neonatal Intensive Care Unit, Mansoura University Children's Hospital, Gomhoria Street, Mansoura 35516, Egypt

## Abstract

Patent ductus arteriosus (PDA) is a common clinical condition in preterm infants. Preterm newborns with PDA are at greater risk for several morbidities, including higher rates of bronchopulmonary dysplasia (BPD), decreased perfusion of vital organs, and mortality. Therefore, cyclooxygenase (COX) inhibitors and surgical interventions for ligation of PDA are widely used. However, these interventions were reported to be associated with side effects. In the absence of clear restricted rules for application of these interventions, different strategies are adopted by neonatologists. Three different approaches have been investigated including prophylactic treatment shortly after birth irrespective of the state of PDA, presymptomatic treatment using echocardiography at variable postnatal ages to select infants for treatment prior to the duct becoming clinically significant, and symptomatic treatment once PDA becomes clinically apparent or hemodynamically significant. Future appropriately designed randomized controlled trials (RCTs) to refine selection of patients for medical and surgical treatments should be conducted. Waiting for new evidence, it seems wise to employ available clinical and echocardiographic parameters of a hemodynamically significant (HS) PDA to select patients who are candidates for medical treatment. Surgical ligation of PDA could be used as a back-up tool for those patients who failed medical treatment and continued to have hemodynamic compromise.

## 1. Introduction

The incidence of patent ductus arteriosus (PDA) in preterm infants varies between 40% and 60% on the third day of life, depending on the estimated gestational age [1–4]. There is no consensus among neonatologists on the management of PDA. The reason for this variation is that current evidence does not mandate one treatment over any other. In this review we are going to find answers, based on the best available evidence, for three main questions regarding PDA management: to treat or not to treat? when to treat? and how to treat?

## 2. To Treat or Not to Treat?

In recent years there has been a growing debate about whether or not to treat a persistent PDA in neonates. The preterm PDA has shifted from being viewed as a pathologic condition causing morbidities and mortality in the preterm infant to being proposed as an innocent physiological bystander [1–8].

### 2.1. Evidence for Treatment

#### 2.1.1. Association between PDA and Neonatal Morbidities and Mortality

The association between PDA and common neonatal morbidities and mortality is the main reason why neonatologists tried to close the PDAs for decades. A ductal left-to-right shunt will cause increased pulmonary blood flow. In preterm infants with respiratory distress syndrome, who exhibit low plasma oncotic pressure and increased capillary permeability, a PDA can result in an interstitial and alveolar pulmonary edema and decreased lung compliance. This, in turn, will lead to higher ventilator settings prolonged ventilation with potentially high oxygen load [9] and increase the probability of BPD. Furthermore, PDA was found to be associated with worsening pulmonary disease [10] and pulmonary hemorrhage [11]. In a large prospective multicenter study, PDA in ventilated very low-birth-weight (VLBW) infants was associated with increased risk of BPD regardless of the timing of the diagnosis of PDA with an odds ratio (OR) of 1.9 [12]. PDA has also been considered an independent risk factor for the development of necrotizing enterocolitis (NEC) with an OR of 1.8 [13]. In addition, myocardial dysfunction due to left-sided volume overload together with a ductal steal phenomenon will worsen systemic perfusion. PDA was found to be an independent risk factor for inotrope-resistant hypotension [14], impaired renal function [15], intraventricular hemorrhage (IVH) [16], and periventricular leukomalacia (PVL) [17] in preterm infants. Moreover, PDA is associated with 4- to 8-fold increase in the mortality of preterm infants [5, 18]. The evidence for some of these associations is conflicting and does not imply causation. It is unclear if these morbidities are a result of the left-to-right PDA shunt, PDA treatment, or consequences of prematurity [19]. A recent retrospective study adds further evidence that PDA has no significant effect on mortality and major morbidities in VLBW infants [20].

#### 2.1.2. Biologic Plausibility

Doppler ultrasonographic studies have demonstrated impaired cerebral blood flow (CBF) in preterm infants with a PDA suggesting a role in the pathogenesis of IVH [16]. Moreover, the reduced abdominal aorta and superior mesenteric artery blood flow “diastolic steal” in preterm infants with PDA may contribute to the development of NEC [21–23].

#### 2.1.3. Delaying Pharmacologic Treatment Is Associated with Decreased Response to COX Inhibitors

In preterm infants, the ductal tissue matures and becomes less regulated by prostaglandins with advancing postnatal age [24, 25]. Accordingly, delaying pharmacologic treatment decreases response to COX inhibitors resulting in lower success rate thereby increasing the rate of surgical ligation.

#### 2.1.4. Permissive Tolerance of PDA May Increase the Risk for BPD

A recent study enrolling 129 preterm infants (501–1500 g birth weight) in 4 different NICUs in the Netherlands has demonstrated that the BPD rate and the combined outcome of death after day 7 and/or BPD was higher in the period where permissive tolerance of PDA was applied (fluid restriction and watchful waiting for PDA closure, limiting indomethacin, or surgical ligation to only those infants with large PDAs needing significant respiratory support) compared to the period where traditional management with indomethacin and/or surgical ligation used early to close PDAs. However this was not associated with significant changes in other neonatal morbidities [26].

### 2.2. Evidence against Treatment

#### 2.2.1. High Rate of Spontaneous Closure of PDA

Functional closure of the ductus arteriosus occurs in almost 50% of full-term infants by 24 hours, in 90% by 48 hours, and in all by 72 hours after birth. In healthy preterm infants of ≥30 weeks' gestation, duct closure occurs by the fourth day after birth, while preterm infants of <30 weeks' gestation, with severe respiratory distress, have a 65% incidence of PDA beyond the fourth day of life [27–31].

A prospective study demonstrated a spontaneous closure of the PDA in the first 10 days of life in at least 35% of extremely low-birth-weight (ELBW) infants and up to 70% in neonates of >28 weeks' gestation. There was a direct relationship between gestational age and spontaneous closure and for each additional week above 23 weeks, the odds of spontaneous closure increased by a ratio of 1.5 [28]. Among infants of <27 weeks' gestation with a persistent PDA at the time of hospital discharge, 75% of the infants will spontaneously close their PDA by the end of the first year [32].

#### 2.2.2. Nonefficacy of Treatment

Clinical trials have failed to demonstrate a meaningful long-term advantage of therapeutic intervention for the ductus arteriosus. Meta-analyses of RCTs have not demonstrated any beneficial effect for early PDA closure on neonatal morbidities including BPD, NEC, neurosensory impairment, death, or the combined outcomes of death or BPD and death or neurosensory impairment [6, 33–38]. Although indomethacin prophylaxis was associated with a reduced risk of IVH or IVH > grade II, this did not result in better long-term neurodevelopmental outcomes [39–41].

#### 2.2.3. Side Effects of Treatment

COX inhibitors have many systemic side effects, as they constrict not only the ductus arteriosus but also the arteries that supply blood to the heart, brain, kidneys, and gut. Indomethacin produces significant reductions in renal [42], mesenteric [43], coronary [44], and cerebral blood flow and reduces cerebral oxygenation [45]. Treatment with COX inhibitors may be associated with transient renal impairment [46], and this effect is more pronounced with indomethacin [47]. Treatment with COX inhibitors is a risk factor for spontaneous intestinal perforation in VLBW infants [48, 49], especially when used in conjunction with corticosteroids [50]. Concerns were also raised on the possible interference of ibuprofen on the albumin-bilirubin binding [51].

Not only are surgical interventions to close the ductus arteriosus associated with additional short-term complications [52–61], but also concerns have been raised about its long-term complications. Early surgical ligation has recently been shown to be an independent risk factor for the development of BPD [62], and it impedes lung growth [63]. Additional data have indicated that infants whose ductus arteriosus is ligated may be at a greater risk for poor neurodevelopmental outcome, BPD, and severe retinopathy of prematurity (ROP) as compared to medically treated infants [58, 64]. A recent study has demonstrated that surgical ligation of PDA in preterm neonates was associated with increased neonatal mortality/morbidity in all analyses adjusted for measured confounders that attempt to account for treatment selection bias [65]. These data suggest that there are, at least, some patients who are unnecessarily exposed to the adverse effects of therapeutic intervention. The risk-benefit balance for these interventions is therefore unknown.

### 2.3. Individualized Approach

Most neonatologists agree that not all PDAs require treatment, but definitely there is a subgroup of PDA that should be closed. There is individual variability among preterm infants, affecting who will respond to which drug and which dose. It is of paramount importance to predict the responders versus the nonresponders, those who will close after repeated courses and those who will close only if given a higher dose. Several studies have tried to define the target population that should be treated using different clinical, echocardiographic, pharmacokinetic, and biochemical parameters.

#### 2.3.1. Clinical Parameters

Lower birth weights and gestational ages have been associated with pharmacologic treatment failures and eventual need for surgical ligation [66]. Moreover, gestational age is an important risk factor for IVH; thus some clinicians recommend indomethacin prophylaxis for extremely immature babies (23–25 weeks) to prevent IVH [4]. More aggressive approach to PDA should be considered in the presence of chorioamnionitis and/or sepsis, because sepsis not only reduces the probability of spontaneous closure but can also induce the reopening of an already closed duct [4]. Other clinical parameters include exposure to antenatal steroids, the absence of significant respiratory distress, and postnatal age at the time of treatment.

#### 2.3.2. Echocardiographic Parameters

Echocardiography is widely used to define HS-PDA requiring treatment and to exclude duct-dependent congenital heart disease. Although there are no stringent echocardiographic criteria to define the need for therapeutic intervention, several echocardiographic parameters have been correlated with PDA hemodynamic significance and with therapeutic responsiveness. These parameters include: PDA diameter >1.4 mm, the internal diameter of the ductus/body surface area ratio, a low-velocity pulsatile flow pattern, left atrium to aorta ratio >1.4, and diastolic reverse flow in the aorta, mesenteric, cerebral, and renal arteries [67–71]. Longitudinal echocardiographic assessment of PDA shunt flow pattern can reflect the hemodynamic changes in PDA after initial dose of COX inhibitors and predict the need for treatment accurately [71, 72]. McNamara and Sehgal [73] proposed a staging system for the severity of PDA based on echocardiographic and clinical parameters. There is evidence that this staging system facilitates the identification of preterm infants at increased risk of respiratory morbidity [74]. The rates of referral for PDA ligation have been reduced by over 50% after the introduction of this scoring system to the NICU care at the Hospital for Sick Children, Toronto, which may be due to the avoidance of intervention in borderline cases of PDA [75].

#### 2.3.3. Pharmacokinetic Parameters

Few studies have evaluated individualizing COX inhibitors dosing based on targeting “therapeutic” plasma concentrations. Al Za'abi et al. [76] failed to demonstrate any dose-response relationship between varying plasma indomethacin concentrations and ultimate PDA closure [76]. On the other hand, trough serum ibuprofen concentrations on the first treatment day seems to be an important factor for a successful ductal closure [77].

#### 2.3.4. Biochemical Parameters

Biomarkers such as B-type natriuretic peptide (BNP), aminoterminal B-type natriuretic peptide (NT-proBNP), and cardiac troponin T (cTnT) may be used to identify significant PDA and determine indication, timing, and treatment options [78]. They are of particular benefit where point of care echocardiography is not available particularly if they are combined with clinical evaluation.

Plasma BNP correlated with magnitudes of the ductal shunt [79–81]. Hsu et al. [82] suggested that high baseline BNP concentrations may be predictive of poor response to indomethacin and of increased need for surgical ligation of the PDA. Mine et al. [83] used the maximal value of blood BNP within the first 5 days of life as a predictor for the need to surgical ligation of PDA and the cut-off value was estimated to be 2000 pg/mL. Attridge et al. [84] demonstrated that BNP-guided therapy (i.e., no indomethacin administration if the BNP concentration is <100 pg/mL within 12 or 24 hours after the first dose) reduced the number of indomethacin doses during the first course of treatment; therefore, it may reduce the side effects of indomethacin.

Both NT-pBNP [85] and cTnT concentrations [86] increase in the presence of a HS- PDA, correlating with echocardiographic markers, and fall following successful treatment. Plasma NT-pro-BNP concentrations were found to be good indicators of HS-PDA [80, 87]. Moreover, plasma NT-pro-BNP and cTnT concentrations were higher in preterm infants with a PDA who subsequently develop IVH grade III/IV or death [79]. On the other hand, a more recent study found no differences in baseline NT-proBNP concentrations between those who responded and those who did not respond to medical treatment, and they also found that the decrease in NT-proBNP concentrations after treatment did not correlate with treatment success or failure [81].

In an interventional study in preterm infants <33 weeks' gestation, indomethacin therapy was given if plasma NT-proBNP concentration was ≥10,180 pg/mL on the 2nd day of life, the cutoff for predicting HS-PDA. On day 2, 19 (38%) infants had plasma NT-proBNP above the cutoff and received indomethacin therapy; none of them developed later HS-PDA, while 1 of 31 infants with NT-proBNP below the cut-off level developed clinical HS-PDA. Overall, no enrolled infants had either reopening of ductus or PDA ligation [88].

Recently, urinary NT-proBNP was found to be a simple and noninvasive alternative. Urinary NT-proBNP/creatinine ratios on day 14 were higher in 14 ventilated infants who did not respond to pharmacological treatment and subsequently required surgical PDA closure than in ventilated infants with successful pharmacological PDA closure [89].

## 3. When to Treat?

Timing of PDA treatment has gained the interest of scientists over the past years. Early intervention for asymptomatic duct carries the benefit of higher success rate but increases the risk of over exposure, while late intervention for symptomatic duct minimizes overexposure but increases the risk of treatment failure and surgical ligation. Three different strategies have been investigated including prophylactic treatment shortly after birth without reference to the state of PDA, presymptomatic treatment using echocardiography at variable postnatal ages to select infants for treatment prior to the duct becoming clinically significant, and symptomatic treatment once PDA becomes clinically apparent or hemodynamically significant.

### 3.1. Prophylactic Therapy

The decline of the pulmonary vascular resistance and pulmonary artery pressure occurs earlier in preterm infants, usually after the first 24 hours of life [90]; thus most of the preterm infants have clinical signs of PDA within the first 2 or 3 days [91]. Accordingly, the window of opportunity for prophylactic therapy for PDA involves giving treatment within the first few days, particularly the first 24 hours, of life.

When first introduced, predefined gestation or weight-based criteria for prophylactic therapy were needed to minimize unnecessary exposure. Most of the conducted researches on prophylactic therapy included preterm infants <32 weeks' gestation and VLBW infants <1500 grams [92–98].

Mahony et al. [99] studied the effect of prophylactic indomethacin in preterm infants <1700 grams at a mean age of 2.9 days and found that infants weighing >1000 gram have a higher chance for spontaneous closure concluding that prophylactic indomethacin is more beneficial in ELBW infants less than 1000 grams. Extended 3 to 6 days' course of low-dose (0.1 mg/kg/d) indomethacin has been widely accepted as the conventional prophylactic course [100]. A trial of using an escalating higher dose (0.2 or 0.5 mg/kg/d) of indomethacin was found to have a little effect on the rate of PDA closure but was associated with higher rates of moderate/severe ROP and renal compromise [101]. The efficacy of prophylactic indomethacin on short and long term neonatal outcome has been well investigated. Earlier studies have shown that prophylactic indomethacin decreases the subsequent incidence of symptomatic PDA and IVH in preterm infants [94, 99, 100]. However, later studies did not show beneficial effect of prophylactic indomethacin on the rate of survival or long-term disability [102, 103]. Fowlie et al. [34, 104] conducted a meta-analysis on 19 trials of prophylactic indomethacin therapy and found that prophylactic indomethacin has short-term benefits including reduction in symptomatic PDA, the need for duct ligation, and severe IVH with no evidence of either benefit or harm on neurodevelopmental outcome. More recently, prophylactic indomethacin was shown to decrease cerebral perfusion which may be harmful to the developing brain [105] and was found to worsen the short-term respiratory outcomes in ELBW infants [106].

Prophylactic ibuprofen therapy at a dose of 10 mg/kg in the first 24 hours of life followed by 5 mg/kg after 24 hours and 48 hours did not show any superiority over prophylactic indomethacin therapy. In a meta-analysis of seven studies comparing prophylactic ibuprofen with placebo, prophylactic ibuprofen was found to decrease the incidence of PDA on day three, decreased the need for rescue treatment with indomethacin, and decreased the need for surgical ligation [107]. However, the use of prophylactic ibuprofen negatively affected the renal function of preterm infants with no significant differences in mortality, IVH, or BPD [107]. On the other hand, two trials on oral ibuprofen had similar results but showed an increased risk of gastrointestinal bleeding [108, 109]. Accordingly, authors concluded that prophylactic ibuprofen exposes many infants to renal and gastrointestinal side effects without any important short-term benefits and is not recommended [107]. A recent study compared prophylactic versus expectant ibuprofen for asymptomatic PDA and found that infants with mild signs of PDA do not benefit from prophylactic ibuprofen compared with delayed treatment [110].

Based on the above evidence of nonbeneficial short-term effect and the absence of long-term benefits, the use of prophylactic indomethacin or ibuprofen practice has been abandoned by most neonatologists.

### 3.2. Presymptomatic Therapy

The concept of presymptomatic treatment of PDA was to restrict the use of therapy to a group of infants with an asymptomatic duct, rather than treating all preterm infants prophylactically, getting a greater chance of benefit together with limiting the possibility of significant side effects.

In a meta-analysis of 3 trials, it was reported that presymptomatic treatment of PDA reduced the incidence of symptomatic PDA and duration of supplemental oxygen with no effect on the rate of mortality, BPD, IVH, ROP, or length of ventilation [33]. The three involved trials in this meta-analysis did not report any long-term neurodevelopmental outcomes for their studied infants [33]. Van Overmeire et al. [111] compared early (day 3) to late (day 7) indomethacin treatment in infants with echocardiographically diagnosed moderate or severe PDA and found that early treatment was associated with more renal side effect but without any evidence of respiratory advantage or any difference in other clinical outcomes.

Presymptomatic treatment, within 72 hours of life, of PDA with intravenous (IV) ibuprofen was found to be effective in the early closure of PDA in preterm neonates with a trend toward decreased PVL. However, other outcomes including death, IVH, NEC, daily fluid intake/output, liver function, BPD, and ROP did not differ [112].

According to the above evidence, presymptomatic indomethacin or ibuprofen therapy for PDA in preterm infants is not recommended.

### 3.3. Symptomatic Therapy

Expectant approach of treating PDA at a later time, only when signs indicate hemodynamic significance, allows for possible spontaneous closure. It carries the advantage of minimizing the risk of exposure of preterm infants to the hazards of therapy but the disadvantage of late intervention such as lower success rate and increased exposure of preterm infants to the hazards of HS-PDA. One of the challenges of expectant symptomatic approach is to clearly define a HS-PDA as discussed earlier. Gersony et al. [113] randomized 421 preterm infants with HS-PDA into three groups of intervention: conservative with indomethacin, conservative then back-up indomethacin, and conservative then surgery. They found that administration of indomethacin concurrent with or as back-up to usual medical therapy at the time of diagnosis resulted in a significant increase in the rate of ductal closure compared to conservative treatment. Although mortality did not differ significantly, infants given indomethacin as a back-up to conservative therapy had a lower incidence of bleeding than those to whom indomethacin was given with initial conservative therapy. They concluded that administration of indomethacin only when conservative treatment fails appears to be the preferable approach for the management of symptomatic PDA in preterm infants.

Only one clinical trial has compared late indomethacin therapy with continued medical management without indomethacin and found a significant decrease in the rate of mechanical ventilation with a trend toward decreasing NEC and ROP in the indomethacin-treated group. However, the sample size of this trial was too small to make any firm conclusions [91].

As the available evidence does not support prophylactic or presymptomatic approach for PDA, expectant symptomatic intervention for HS-PDA seems to be the most reasonable approach of care. Further studies are needed to evaluate the validity of expectant symptomatic therapy compared to conservative treatment.

## 4. How to Treat?

### 4.1. Conservative Management

Although fluid restriction has been widely recommended in management of PDA [113–116], its benefits to hazards have not been assessed systematically. Fluid restriction may decrease circulating blood volume and the overload of the pulmonary circulation that in turn may improve the respiratory function [117]. In VLBW infants, retrospective studies have shown an association between increased fluid intake during the first week of life with a lack of appropriate physiologic weight loss and increase in the incidence of BPD. Although restricted fluid intake in the first few days of life is associated with a decreased incidence of PDA and BPD, a meta-analysis has shown that fluid restriction may result in a decrease in left ventricular output through a decrease in left ventricular preload that in turn may cause a reduction in systemic blood flow [118]. In a recent prospective observational study on 18 VLBW infants with HS-PDA, fluid intake was restricted to 100–120 mL/kg/d. The authors found that fluid restriction did not change blood gas values, O2 requirements, PDA diameter, systemic blood pressure, and flow velocity in the PDA, left pulmonary artery, or the left atrium. However, it was associated with decreased blood flow in the superior vena cava and superior mesenteric artery [117].

There is no enough evidence to support the routine use of diuretics for prevention or treatment of congestive heart failure in HS-PDA, and a systematic review of the coadministration of furosemide in indomethacin-treated neonates showed a trend toward failure of ductal closure in furosemide-treated patients because furosemide increases prostaglandins production and could potentially decrease the ductal response to indomethacin [119]. In a study of 68 preterm infants treated with indomethacin and were randomly assigned to receive furosemide (1 mg/kg) or placebo, there was no difference in the rate PDA closure, neonatal morbidity, or mortality in the furosemide-treated group [120].

Oxygen therapy has been proposed in the pathogenesis of duct closure in preterm infants. In a retrospective study including 263 ELBW infants, infants treated with lower oxygen saturation target range policy (83–89% versus 89–94%) had more incidence of HS-PDA; however, none of these infants required surgical ligation later on [121].

### 4.2. Pharmacological Treatment (Table 1)

#### 4.2.1. Indomethacin

Indomethacin is the most widely used nonselective COX inhibitor for PDA closure. In a large national collaborative trial involving 421 preterm infants (<1750 grams) with HS-PDA, duct closure was observed in 79% in indomethacin-treated infants versus 35% with placebo [113]. Van Overmeire and Chemtob [122] reported a closure rate of 70%–90% in HS-PDA with indomethacin; however, 13%–53% of cases relapsed or remained open after treatment. The closure rate of PDA with indomethacin is dependent on birth weight of preterm infants. Gersony et al. [113] reported a postindomethacin closure rate of 80% to 86% in infants weighed 1000–1750 g and 54% in infants less than 1000 g.

Several dosing regimens of indomethacin have been used for prophylaxis and treatment of PDA [123]. The most commonly used prophylactic regimen includes 3 to 6 IV doses of 0.1 mg/kg every 24 hours, whereas treatment usually involves an initial dose of 0.2 mg/kg followed by two doses of 0.1-0.2 mg/kg every 12 hours [99, 124, 125]. In cases of failure or relapse following initial therapy, a second course was found to successfully close the PDA in up to 44% of cases [126]. Although most clinicians have to try more than one course of indomethacin before moving to surgical ligation, this approach has not been evaluated in controlled trials [123].

Five RCTs were included in a meta-analysis comparing prolonged (>4 doses) and short course (<4 doses), there was no significant difference between PDA closure, need for ligation, reopening or retreatment, mortality, BPD, or IVH. There was an increased incidence of NEC [number needed to harm (NNH) = 13] and renal impairment (NNH = 6) [127] in the prolonged course group.

Sperandio et al. [128] reported that high-dose indomethacin up to 1 mg/kg once resulted in an overall closure rate of 98.5%. There was no difference in the incidences of renal or electrolyte abnormalities, gastrointestinal bleeding, IVH, and PVL when high dose was compared with the conventional one. Jegatheesan et al. [129] had tried higher doses of indomethacin after failure of conventional doses and they found little effect on duct closure together with more adverse effects.

Intravenous indomethacin is usually given as bolus over 30 min, only two small trials compared continuous with intermittent bolus administration and reported no statistically significant differences in PDA closure at day 2 or day 5, rates of reopening of PDA, neonatal mortality, IVH, and NEC [130].

Adverse effects might occur frequently during indomethacin treatment including hyponatremia, oliguria, active bleeding, and impaired renal function, which are transient and seem to have no long-term sequelae. NEC (stage II and III), IVH, and focal gastrointestinal perforation are rarely found during therapy. These side effects of indomethacin are due to the nonselective vasoconstrictive effect of the drug and the reduction of blood flow through various organs [131, 132].

Indomethacin is contraindicated in preterm infants with the following: proven or suspected life-threatening infection that is untreated, active bleeding especially gastrointestinal or intracranial, coagulation defects and/or thrombocytopenia, significant impairment of renal function, suspected or proved NEC, and PDA-dependent congenital heart diseases [133].

#### 4.2.2. Ibuprofen

Ibuprofen, another COX inhibitor, is effective in closing PDA without reducing cerebral, intestinal, or renal blood flow [42, 134, 135]. In addition, ibuprofen was found to augment CBF autoregulation and has neuroprotective effects following exposure to oxidative stress in a piglet model [136, 137]. The rate of PDA closure in preterm infants varies considerably with multiple courses of Ibuprofen. Studies have shown a rate of closure between 45 and 92% after the first course, 40 and 54% after the second course, and 19 and 66% after the third course [138–140]. Differences in closure rates between the studies are probably due to differences in their designs and methodology.

The initial dosing guidelines consist of 10 mg/kg loading dose followed by 5 mg/kg/d every 24 hours twice, total of 3 doses in 3 days [141, 142]. A higher dose regimen (20-10-10 mg/kg) might achieve a higher closure rate but must be balanced with the tolerability and safety [142]. A recent RCT demonstrated that the high-dose regimen is more effective than the standard-dose regimen in closing PDA in preterm infants <29 weeks' gestation without increasing the adverse effect rate [143].

Oral ibuprofen is an alternative for the treatment of PDA. Oral ibuprofen was more effective than IV ibuprofen (84.6% versus 62%) for ductal closure in VLBW infants. Oral, but not IV, ibuprofen was associated with rise in cystatin-C levels, a marker of impaired renal function, in preterm infants indicating that infants with borderline renal function may need careful monitoring [144]. Erdeve et al. [145] performed an RCT of oral versus IV ibuprofen in 80 preterm infants and found a higher initial closure rate and reduction in the incidence of BPD with oral ibuprofen though there was a higher reopening rate in infants who received this treatment. A meta-analysis that included two studies (*n* = 166) comparing oral ibuprofen with IV ibuprofen showed higher PDA closure rate with oral ibuprofen in comparison with IV ibuprofen and did not show a significant difference in adverse effects [146].

Ibuprofen therapy, particularly the oral form, was found to be associated with adverse events mainly on gastrointestinal tract [147]. Spontaneous intestinal perforation with oral administration of ibuprofen for PDA was previously reported [148], despite the evidence of preserved renal and mesenteric tissue oxygenation [149]. Intravenous ibuprofen lysine has been reported to be safe, while ibuprofen-THAM is associated with increased risk for NEC [92]. Although ibuprofen theoretically inhibits platelet adhesiveness, Dani et al. [96] observed no differences in serial platelet function and number between placebo and ibuprofen. Ibuprofen is 99% protein bound which may displace bilirubin from albumin binding sites [150]. In vitro studies have demonstrated that ibuprofen displaces bilirubin from albumin and increases the plasma levels of unbound bilirubin [151]. However, in vivo studies did not show similar effects in preterm infants treated by the current recommended doses of 10-5-5 mg/kg/day particularly if total bilirubin levels were below 10 mg/dL before treatment [152, 153]. In the French collaborative trial, three neonates developed pulmonary hypertension when given ibuprofen THAM [92], all these infants responded to inhaled nitric oxide, and this complication was not reported with IV ibuprofen-lysine [154].

Accordingly, ibuprofen is contraindicated in treatment of PDA in preterm infants with renal failure, hyperbilirubinaemia, gastrointestinal perforation, severe thrombocytopenia [155], life-threatening infections, known or suspected NEC, duct-dependent congenital heart disease, and hypersensitivity to ibuprofen [156].

Studies comparing ibuprofen to indomethacin therapy showed similar efficacy of both drugs for PDA closure, less nephrotoxic effects of ibuprofen, less adverse peripheral vasoconstrictive effects for ibuprofen, with no difference in mortality, IVH, and BPD [157]. Two recent meta-analyses have demonstrated that both IV indomethacin and ibuprofen are equally effective in closing PDA [158, 159]. Long-term neurodevelopmental outcome studies on ibuprofen are not available and are needed.

#### 4.2.3. Paracetamol

Recently, paracetamol has been shown to be an alternative treatment for closure of PDA because of its safety profile and low cost. The effect of paracetamol is through prostaglandin synthetase inhibition; this action is at the peroxidase segment of the enzyme. Peroxidase is activated at tenfold lower peroxide concentrations than COX. Therefore, paracetamol-mediated inhibition is facilitated at reduced local peroxide concentrations. This would permit peroxidase inhibition to be optimally effective under conditions in which COX inhibition is less active [160, 161].

Hammerman et al. [162] reported that oral paracetamol (15 mg/kg per dose/6 hrs for 3 days) was effective in closing the HS-PDA in 5 VLBW infants (2 infants who did not respond to ibuprofen and to 3 infants with contraindications to ibuprofen). Another study reported that a similar dose of oral paracetamol was effective in closing the PDA in five (71.4%) of seven preterm infants in whom ibuprofen treatment was unsuccessful [163]. Yurttutan et al. [164] reported successful closure of PDA in five (83.3%) of six preterm infants treated with oral paracetamol as a first-line drug in the medical management of PDA. Intravenous paracetamol is also an alternative option in patients in whom feeding is contraindicated or have feeding intolerance. Successful PDA closure was observed in 83.3% and 100% of VLBW infants who ranged in gestational age from 24 to 32 weeks [165, 166]. Recently, it was reported that IV paracetamol may increase transaminases concentration in preterm infants and that a lower dose of paracetamol is effective [167]. Prospective comparative trials are urgently needed to establish both the effectiveness and safety data of paracetamol when used for PDA closure.

### 4.3. Surgical Ligation oF PDA

In 1963 surgical PDA ligation was first performed in a preterm infant [168]. Since then many authors suggested safety and effectiveness of surgical ligation in the preterm infant [131, 169–171]. Surgical closure of the PDA implies application of either suture ligatures or vascular clips [57]. Video-assisted thoracoscopic PDA ligation was reported to provide a less disturbing alternative to the usual surgical approach [172].

Although surgical closure of PDA in preterm infants is considered a well-tolerated procedure, many short-term complications were identified including unilateral vocal cord paralysis [52–55], diaphragmatic paresis [56] or eventration [57], intraoperative bleeding, chylothorax [55, 57, 58], pneumothorax [173], cardiorespiratory decompensation in the immediate postoperative period [59, 60], relative adrenal function insufficiency [174], and scoliosis [61]. Furthermore, there is controversy regarding association of NEC with surgical ligation [32, 173, 175].

Several retrospective studies demonstrated lack of pulmonary benefit from surgical ligation of PDA and even increased risk for BPD [58, 64, 176, 177]. A prospective study revealed similar rates of death, BPD, ROP, and IVH in infants treated by prophylactic surgical PDA ligation on the first day of life to those treated by medical therapy (not including indomethacin) [175]. Reanalysis of data from this trial, considering BPD definition as oxygen requirement at 36-week postmenstrual age, showed that patients in the prophylactic ligation group had a higher rate of BPD [62]. A recent Cochrane review [173] compared effect of surgical ligation of PDA with medical treatment with COX inhibitors as an initial therapy and included analysis of only one trial [113]. The results revealed no statistically significant difference between the two groups as regards occurrence of BPD. However ROP occurred more frequently in the surgical group compared to the indomethacin group.

Several observational retrospective studies analyzed the neurodevelopmental outcome of preterm infants with surgically-ligated PDA, in comparison with those who were not subjected to surgical ligation. A secondary analysis of data from TIPP trial revealed increase risk of neurosensory impairment and cognitive delay at 18 months in infants who were subjected to surgical ligation [64]. Further subsequent studies, in which multivariate analysis of data was done to adjust other potential confounders for abnormal neurodevelopmental outcome, revealed that ligation status itself (ligation versus no ligation) does not appear to be a predictor of neurodevelopmental outcome [58, 176, 178]. However, Wickremasinghe et al. [178] found an increased incidence of abnormal neurodevelopmental outcome in patients who underwent ligation before 10 days of age.

There are two approaches for surgical ligation of PDA; the early surgical ligation approach involves surgical ligation as soon as possible for all PDAs that failed to close after indomethacin therapy irrespective of ventilatory requirements or the degree of left-to-right shunt [94], whereas the selective surgical ligation approach considers surgical ligation only if cardiopulmonary compromise develops [32]. Analysis of the data of 216 and 180 infants who were subjected to “early ligation” and “selective ligation” approaches, respectively, in two successive time periods revealed that the rates of occurrence of BPD, ROP, sepsis, and neurologic injury were similar in both groups. In the contrary, the overall rate of NEC was significantly lower in patients treated with selective ligation compared with those treated with early ligation (OR, 0.26; 95% CI, 0.07–0.95) [32]. Neurodevelopmental followup was conducted for 224 of those infants up to 18–36 months of age. The results revealed that unadjusted incidences of abnormal neurodevelopmental outcome were significantly lower in infants treated with selective ligation (OR, 0.07; *P* = 0.046) [178]. Given the limitations of current evidence, appropriately powered RCTs with long-term followup are urgently needed to better delineate the best approach for surgical ligation of PDA.

## 5. Conclusions

**Figure 1:**
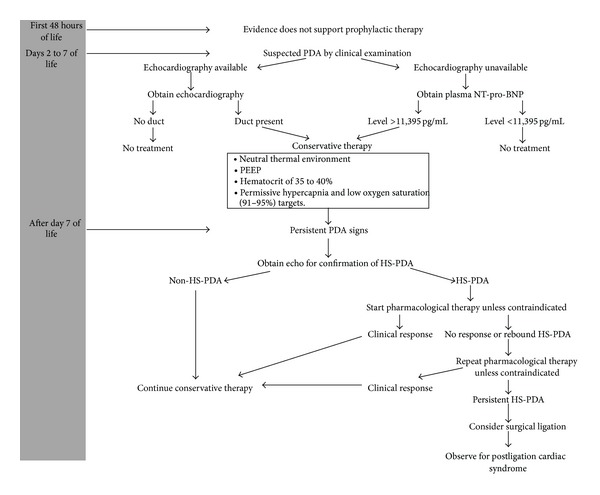
Suggested timeline approach for management of PDA in preterm infants based on the best available evidence.

The decision of treatment of PDA should be individualized, according to clinical, echocardiographic, and biochemical parameters that validate hemodynamic significance of PDA. As the available evidence does not support prophylactic or presymptomatic approach, expectant symptomatic treatment for HS-PDA seems to be the most reasonable approach. A suggested timeline algorithm for timeline management of PDA in preterm infant according to the best available evidence is shown in Figure 1.

Fluid restriction has no beneficial effects on pulmonary or systemic hemodynamics in infants with PDA. Medical management involves the use of COX inhibitors: indomethacin and ibuprofen lysine. They are equally effective in closing the PDA. Infants whose ductus arteriosus is surgically ligated may be at a greater risk for poor neurodevelopmental outcome, BPD, and severe ROP. Until new evidence becomes available, it seems wise to reserve surgical closure for those neonates who have failed medical therapy and have echocardiographic evidence of a large duct or ongoing significant oxygen and ventilatory requirements.

## Figures and Tables

**Table 1:** Pharmacological therapy for PDA in preterm infants.

Drug	Timing	Dose	Route	Duration	Benefits	Limitations	Recommendations
Indomethacin	Prophylactic (within 48 hours of life)	0.1 mg/kg per dose	Intravenous	3 to 5 doses every 24 hours	(1) Reduce symptomatic PDA (2) Reduce the need for duct ligation (3) Decrease severe IVH	(1) Decrease cerebral perfusion (2) Worsen the short-term respiratory outcomes (3) No evidence of benefit on neurodevelopmental outcome	Evidence does not recommend prophylactic therapy (Grade 1B)
	Therapeutic (1) early presymptomatic (before 7 days), (2) late symptomatic (after 7 days)	0.2 mg/kg/dose	Intravenous	3 to 5 doses every 12 hours	(1) Reduce the need for duct ligation (2) Reduce duct-related morbidities (BPD)	(1) Hyponatremia (2) Oliguria (3) Active bleeding (4) Transient impairment of renal function (5) NEC (stage II and III) (6) IVH (7) Focal gastrointestinal perforation	(1) Evidence does not recommend early presymptomatic therapy (2) Evidence supports late symptomatic therapy for HS-PDA in mechanical ventilation dependent infants (Grade 2B)

Ibuprofen	Prophylactic (within 48 hours of life)	10 mg/kg followed by two additional doses of 5 mg/kg given at 24-hour intervals	(1) Intravenous (2) Oral	3 doses every 24 hours	(1) Decrease the incidence of PDA on day three (2) Decreased the need for rescue treatment with indomethacin (3) Decreased the need for surgical ligation	(1) Transient impairment of renal function (2) Increase the risk for hyperbilirubinemia with high doses (3) Inhibit platelet adhesiveness (4) Oral ibuprofen increases the risk of GIT bleeding and NEC (5) No significant differences in mortality, IVH, or BPD	Evidence does not recommend prophylactic therapy (Grade 1B)
	Therapeutic (1) early presymptomatic (before 7 days), (2) late symptomatic (after 7 days)	10 mg/kg followed by two additional doses of 5 mg/kg given at 24-hour intervals	(1) Intravenous (2) Oral	3 doses every 24 hours	(1) Reduce the need for duct ligation (2) Reduce duct-related morbidities (BPD)	(1) Transient impairment of renal function (2) Increase the risk for hyperbilirubinemia with high doses (3) Inhibit platelet adhesiveness (4) Oral ibuprofen increases the risk of GIT bleeding (5) No significant differences in mortality, IVH, or BPD	(1) Evidence does not recommend early therapy (2) Evidence supports late therapy for HS-PDA in mechanical ventilation dependent infants (Grade 2B)

Paracetamol	Prophylactic	No available data	No available data	No available data	No available data	No available data	No available evidence
	Therapeutic	15 mg/kg per dose	(1) Oral (2) Intravenous	12 doses every 6 hours for 3 days	(1) Close HS-PDA	No available data	Evidence from small case series supports its use.
